# Grass Snakes (*Natrix natrix*) as a Reservoir of *Alaria alata* and Other Parasites

**DOI:** 10.3390/pathogens11020156

**Published:** 2022-01-26

**Authors:** Aneta Bełcik, Mirosław Różycki, Weronika Korpysa-Dzirba, Gianluca Marucci, Zbigniew Fafiński, Patrycja Fafińska, Jacek Karamon, Maciej Kochanowski, Tomasz Cencek, Ewa Bilska-Zając

**Affiliations:** 1 Department of Parasitology and Invasive Diseases, National Veterinary Research Institute, Partyzantow Avenue 57, 24-100 Pulawy, Poland; mrozycki@piwet.pulawy.pl (M.R.); weronika.korpysa@piwet.pulawy.pl (W.K.-D.); j.karamon@piwet.pulawy.pl (J.K.); maciej.kochanowski@piwet.pulawy.pl (M.K.); tcencek@piwet.pulawy.pl (T.C.); ewa.bilska@piwet.pulawy.pl (E.B.-Z.); 2 Department of Infectious Diseases, Instituto Superiore di Sanità, Viale Regina Elena, 00161 Rome, Italy; gianluca.marucci@iss.it; 3 Wetlab, Veterinary Diagnostic Laboratory, Piękna Avenue 6, 09-402 Płock, Poland; pwetlab@wetlab.pl (Z.F.); wetlab@wetlab.pl (P.F.)

**Keywords:** grass snakes, parasites, trematodes, *Alaria alata*, environmental risk

## Abstract

The aim of the study was to investigate the occurrence of *Alaria alata* (Goeze, 1782) in fifty-one grass snakes (*Natrix natrix*) collected in Gostynińsko-Włocławski Landscape Park. Each snake was tested for the presence of *A. alata* mesocercariae using the AMT and MSM methods. 18S ribosomal RNA (18S rRNA), cytochrome C oxidase subunit I (COI) and 28S ribosomal RNA (28S rRNA) genes were amplified by PCR and sequenced for the purpose of species identification. Fifty grass snakes were infected with helminths. The molecular characterization of trematodes allowed us to identify *A. alata* in 30 snakes (58.8%), *Conodiplostomum spathula* (Dubois, 1937) in 16 snakes (31.3%), *Strigea falconis* (Szidat, 1928) in 12 snakes (23.5%), and *Neodiplostomum attenuatum* (Linstow, 1906) in 2 snakes (3.9%), while, in 4 snakes (7.8%), the trematodes species could not be identified. Based on the analysis of 18S and COI sequences, *Crenosoma vulpis* (Dujardin, 1845) was identified in four snakes (7.8%), while nematodes collected from three snakes remained unidentified. The tapeworm sample was identified as *Ophiotaenia*. The obtained results indicate that grass snakes are an excellent vector of *A. alata* and may be a potential source of infection for mammals, e.g., wild boars and foxes, which results in an increased risk of alariosis for consumers of raw or undercooked game meat.

## 1. Introduction

**Figure 1:**
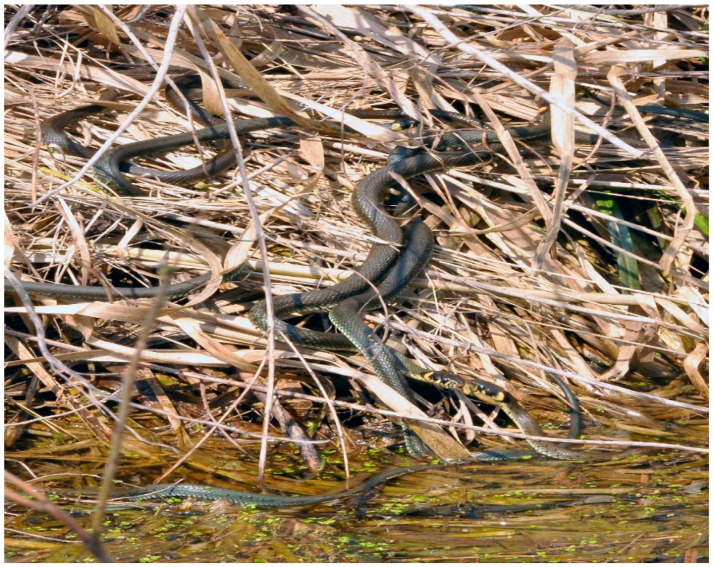
Grass snakes in their typical environment. This photo was taken during the snake’s mating season in April (by T. Cencek).

The European grass snake (*Natrix natrix*) is a non-venomous snake, widely distributed in European countries, from central Scandinavia to Southern Italy, as well as in the Middle East and Northwest Africa. The preferred habitat of grass snakes is woodland and “edge” territories, such as field margins and forest borders (Figure 1). These areas offer adequate refuge and, at the same time, allow thermoregulation by sun-basking. They typically spend the winter season underground, where the temperature is relatively stable [1,2,3], starting brumation in October to November, which lasts until March or April. They lower their body temperature, which causes the heart to hardly beat; air is pumped into the lungs every quarter of an hour. The energy needs are met by a small amount of fat stored in the tail or other parts of the body [3,4,5].

**Figure 2:**
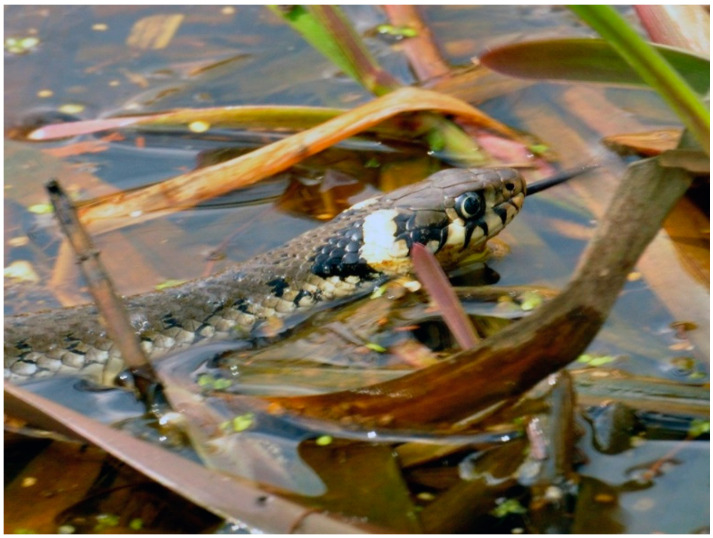
Grass snake—a characteristic yellow spot and an eye with a round pupil are visible (by T. Cencek).

European grass snakes range in length from 1 to 1.5 m; females are usually longer than males. The head is flat with two characteristic crescent-shaped yellow-orange patches on the caudal aspect of the head. The eye is relatively large with a round pupil (Figure 2). The body is the average length and the tail ends with a sharp spike. The skin color may be bright, green and brown. Sexual dimorphism is poorly marked. The only difference is the body length of adult *N.natrix* individuals. Females are longer [6,7]. The prey of grass snakes consists mainly in amphibians, especially the common toad (*Bufo bufo*) and the common frog (*Rana temporaria*), although they may occasionally eat ants and newt larvae. They primarily rely on two senses for detecting prey: vision and olfaction (Jacobson’s organ) [5,8]. Hunting typically occurs along a water’s edge [5,8]. The species has various predators, including corvids (*Corvidae*), storks (*Ciconiidae*), owls (*Strigiformes)*, foxes (*Vulpes* spp.), and even domestic cats (*Felis catus*) [9]. In natural environment, grass snakes are very often the victim of wild boars (*Sus scrofa*) [10].

Free-living snakes may carry a broad range of pathogens, since they are also prey animals. They are involved in the life cycles of various nematodes, trematodes and cestodes, representing an indirect risk of zoonotic infection for humans [11,12]. Historically, about 20 species of parasitic trematodes, nematodes, and cestodes, have been detected in *N. natrix* [1,13,14,15,16,17]. Adult forms of these parasites mainly colonize the gastrointestinal tract, lungs, body cavity and muscle tissue [17]. Grass snakes play the role of paratenic host for the trematode *Alaria alata* (Goeze, 1782) [13].

The life cycle of *A. alata* is complex and includes definitive (wolves, foxes, badgers, martens, lynxes, raccoons, dogs, cats), intermediate (freshwater snails, tadpoles) and paratenic (mainly wild boars, pigs, rodents, martens, ferrets, wild birds and reptiles such as snakes and lizards) hosts [18]. The first intermediate host is a freshwater snail (e.g., *Helisoma, Planorbis* spp.), which becomes infected by miracidia, the *A. alata* hatchling stage. The miracidia develop into sporocysts that produce cercaria, a fast-moving larval stage that emerges from its snail host, penetrates a tadpole and develops into a non-reproductive form, the mesocercaria. The mesocercariae can infect paratenic, as well as definitive, hosts; they are localized in muscle or adipose tissue. The role of the paratenic host is to help the parasite spread, which is of particular importance considering the complex life cycle and the required environmental conditions of this trematode [19,20]. 

The infected snakes facilitate the spread of the trematode to its definitive hosts, as well as to other paratenic hosts [17,18]. The prevalence of *A. alata* in foxes has been demonstrated to be very high in Poland [21]; this has an impact on the occurrence of this trematode in other hosts participating in their life cycle, such as wild boars [22]. Grass snakes can become the prey of wild boars, which are paratenic hosts for *A. alata* [10]. The prevalence of *A. alata* in wild boars has been underestimated for several years, since the parasite was only detected during routine tests of wild boar meat for the presence of *Trichinella* spp. by artificial digestion, which is an improper detection method for this parasite [22]. Recently, the application of the *A. alata* mesocercariae migration technique (AMT) [23,24] revealed that the prevalence of this trematode in the Polish wild boar population is higher than previously believed, highlighting the potential risk of human infection (alariosis) due to infected meat consumption (19).

The main symptoms of alariosis are different, including an increase in circulating eosinophils, as well as an increase in serum immunoglobulin E (IgE). As a result, patients may experience an anaphylactic reaction and a decrease in arterial pressure, leading to vascular collapse and loss of consciousness [11]. Other reactions of the body related to the presence of *A. alata* include inflammation of the intestine, as well as mild respiratory symptoms and retinal nerve collapse (DUSN). Due to the limited number of reports, disease symptoms are specific for different *Alaria* spp. In the worst stage of the disease, alariosis can lead to anaphylactic shock, which may result in death. [11,14,25,26].

The aim of this study was to determine the occurrence of *A. alata* in grass snakes collected from areas where this parasite is also present in wild boars to evaluate its potential role as a paratenic host. Moreover, the characterization of the parasites based on molecular markers such as 18S rRNA, COI and 28S RNA was performed.

## 2. Results

### 2.1. Prevalence the Parasites in a Sample Population of Grass Snakes in “Gostynińsko-Włocławski Landscape Park”

In this study, 50 out of a total of 51 (98.0%) examined grass snakes were infected with helminths. Out of 5158 single parasites detected, the majority of them were trematodes, which were preliminarily assessed as *Alaria* spp. based on their morphology. From 50 grass snakes, one to five parasites from each snake, were taken individually for molecular characterization, and four trematode species were identified, with a different prevalence in the examined snake population. The majority of snakes were infected with only one species of trematodes—*A. alata* was identified in 25/51 snakes (49%), *Conodiplostomum spathula* (Dubois, 1937) was detected in five snakes (9.8%), *Strigea falconis falconis* (Szidat, 1928) in two snakes (3.9%), and *Neodiplostomum attenuatum* (Linstow, 1906) in one snake (1.9%), while trematodes collected in four snakes (7.8%) remained unidentified. Additionally, authors observed mixed infections involving the following species: *C. spathula* and *S. falconis* in seven snakes, *A. alata* with *C. spathula* and *A. alata* with *S. falconis* in two snakes, *C. spathula* and *N. attenuatum* in one snake, and a triple infection with *A. alata*, *C. spathula* and *S. falconis* in one snake. In total, *A. alata* occurred in 30 samples (58.8%), *C. spathula* in 16 (31.4%), *S. falconis* in 12 (23.5%) and *N. attenuatum* in 2 (3.9%) out of all examined grass snakes.

**Figure 3:**
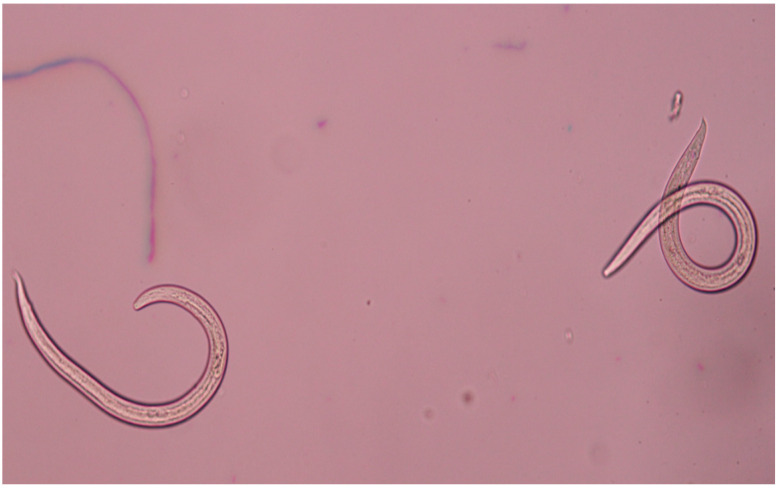
*C. vulpis* specimens observed under a stereomicroscope (magnification 150×) (by E. Bilska-Zając).

Additionally, in seven snakes, nematode specimens were found, four (7.8%) of which were identified as *Crenosoma vulpis* (Dujardin, 1845) (Figure 3), while three remained unidentified. Moreover, in one snake (1.9%), morphologically unidentifiable tapeworm fragments were observed, and molecular analysis allowed us to classify them as belonging to *Ophiotaenia* genera. The highest parasite load was observed in snakes infected with *A. alata*, which in one sample reached the concentration of 365 mesocercariae, while *C. spathula*, *S. falconis*, *N. attenuatum* and *C. vulpis* were present at the maximum level in 15, 4, 2 and 17 specimens, respectively (for detailed information on parasites recovered from each snake, see Table 1).

### 2.2. Molecular Characterizations of Helminths

The molecular characterization, based on partial 18S rRNA sequences, allowed us to identify 30 individuals of *A. alata* (GeneBank accession numbers from OK428859 to OK428892), with 100% identity with accession numbers: MK421337.1, AY222091.1 and HM022225.1, 16 specimens of *C. spathula* (OK248893–OK248918), with 100% identity with accession number: MK089351.1, 12 individuals of *S. falconis* (OK248898–OK248906 and OK428919), with 100% identity with accession numbers: MF628082.1 MF628070.1 MF628072.1, and 2 individuals of *N. attenuatum* (OK428920 and OK428921), with 100% identity with accession number: MG770033.1. Four trematodes remained unidentified because of the low quality of sequences obtained. Regarding the nematodes, four of them were identified as *Crenosoma vulpis* based on COI sequences with 98% identity with homologous GenBank (KM216824) deposited sequences, respectively. Three nematodes remained unidentified due to the low quality of sequences obtained. The 28S rRNA gene was used as a molecular target to identify the tapeworm fragments recovered from one grass snake; BLAST analysis showed 99.17% identity with homologous sequences KP729415.1 of *Ophiotaenia* genera.

## 3. Discussion

The investigation performed on the 51 grass snakes collected in the Gostynińsko-Włocławski Park showed a prevalence of *A. alata* infection of 58.8%. In a similar study conducted in the same area in autumn 2014 [13], in which 15 grass snakes and 1 smooth snake (*Coronella austriaca*) were examined, the *A. alata* prevalence was 81.3%. The two studies demonstrate that this region is characterized by a high prevalence of *A. alata* in snakes. There are older reports on the occurrence of *A. alata* in grass snakes in Poland. The first case was described by Grabda-Kazubska [14], during his investigation into *N. natrix* parasites, in which he observed *A. alata* mesocercariae in 8 out of a total of 12 snakes. Similar studies were conducted by Sulgostowska [1], who identified *A. alata* in all seven of the examined snakes, and by Lewin [15], who observed a prevalence of 46.8% in grass snakes collected in five regions of Poland. Moreover, in 1997, Lewin and Grabda-Kazubska [27], investigating the presence of parasites in *Vipera berus*, detected *A. alata* in 70 out of a total of 152 (46%) examined snakes. All these studies demonstrate that the presence of this trematode in Polish snake populations is not as unusual, since it has been observed for a long time.

Investigations into this zoonotic parasite were also carried out in Belarus, Russia and Romania. In the study of Shimalov and Shimalov [28], conducted between 1980 and 1999, 11 out of a total of 52 (21.2%) tested grass snakes were infected with *A. alata*. In Romania, Mihalca et al. [16] showed the occurrence of mesocercariae in one out of 25 (9.0%) examined grass snakes. In Russia, in the National Park “Smolny”, Kirillov and Kirillova [29] found that 96.7% of the tested grass snakes (n = 91) were infected with *A. alata* mesocercariae. The prevalence of *A. alata* seems to depend on the geographical region. The environment in which grass snakes live is very important for the circulation of this parasite. If the environment is suitable for intermediate hosts (mainly wetlands or their margins) and definitive hosts, the snakes are more likely to become infected [11]. The complex life cycle of *A. alata*, involving many different hosts, makes the occurrence of this parasite strictly dependent on its prevalence in intermediate hosts, as well as on their susceptibility to the infection. The prevalence of *A. alata* (58.8%) found in our study confirms that this parasite is very common in grass snakes from Gostynińsko-Włocławski Park, and thus the local *N. natrix* population can be considered a good reservoir for this parasite, because, in other regions, the prevalence is lower, demonstrating the importance of snakes as a reservoir.

In this study, in addition to *A. alata* the presence of *C. spathula* and *N. attenuatum* trematodes was also observed. According to our knowledge, this is the first report of the occurrence of *C. spathula* and *N. attenuatum* in grass snakes in Poland. The life cycle of these parasites includes freshwater snails as a first intermediate host, and amphibians as a second intermediate host. The adult larval form occurs in the intestines of birds of prey, such as buzzards (*Buteo buteo*) or peregrine falcons (*Falco peregrinus*). Reptiles and mammals appear in the host chain as paratenic hosts for these trematodes. Komorova et al. [30], in research conducted in Slovakia, found *C. spathula* in one out of six (16.7%) tested imperial eagles, as well as in mixed infection with 15 individuals of S. falconis. In the Czech Republic, Sitko [31] reported that 27% of analyzed common buzzards were infected by *N. attenuatum*. These investigations confirm that grass snakes are a common vector for both trematodes and can play an important role in spreading these parasites.

The present study is also, to the best of our knowledge, the first report of *S. falconis* in grass snakes in Poland confirmed by molecular analysis. The observation of 12 snakes infected with *S. falconis* confirms the results previously obtained by Lewin [15], who, based on morphology, identified *S. falconis* in 8 out of a total of 62 (12.9%) examined grass snakes. Additionally, *S. falconis* was also found in vipers (*Vipera berus*) in four different Polish regions [27]. The authors identified this species in four out of a total of 152 (2.63%) vipers collected in the Bieszczady Mountains. This trematode species occurs in birds of prey such as buzzards, hawks and harriers [31,32,33]. Sitko [31] noted the occurrence of *S. falconis* in 27% of the investigated common buzzard (*Buteo buteo*) specimens. The study of [29] Komorova et al. showed a prevalence ranging from 2.7% to 75% in tested birds of prey: buzzards, northern goshawk (*Accipiter gentilis*) and marsh harrier (*C. aeruginosus*). Additionally, the authors reported a few cases of mixed infections involving both *S. falconis* and *N. attenuatum* [29].

In four snakes, we found the nematode *C. vulpis.* This lungworm is a species that often infects wild and domesticated canids [34,35,36] in Europe. Its life cycle includes intermediate hosts, such as snails and slugs, in common with grass snake parasites. The prevalence of this nematode is closely related to the presence of wetlands necessary for the development of larval forms. To the best of our knowledge, there are no reports on the presence of *C. vulpis* in grass snakes, nor in other snake species in Poland. However, according to the life cycle of this nematode, it can be assumed that snakes are vulnerable to infection and may play a role as paratenic hosts [37,38].

The presence of tapeworms was detected in one snake. Morphological identification was not possible because of the poor state of preservation; however, molecular investigation allowed us to assign them to the *Ophiotaenia* genera. The genus *Ophiotaenia* has already been detected in grass snakes. Santoro et al. [39] found *Ophiotaenia europaea* in 93% of examined grass snakes in Italy; Amman et al. [40] detected *Ophiotaenia gilberti* in *Thamnodynastes pallidus* (*Serpentes: Colubridae*) in Paraguay.

## 4. Materials and Methods

### 4.1. Preparing of Samples

**Figure 4:**
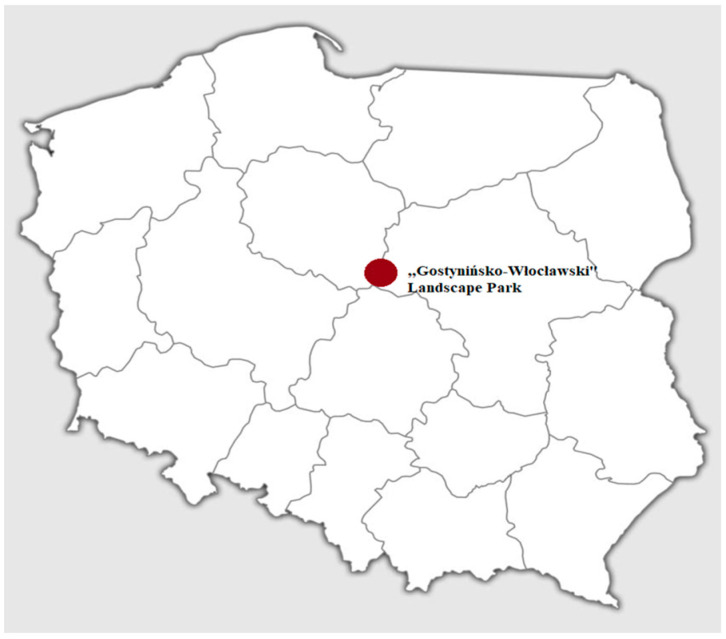
Geographical localization of the Gostynińsko-Włocławski Landscape Park.

**Figure 5:**
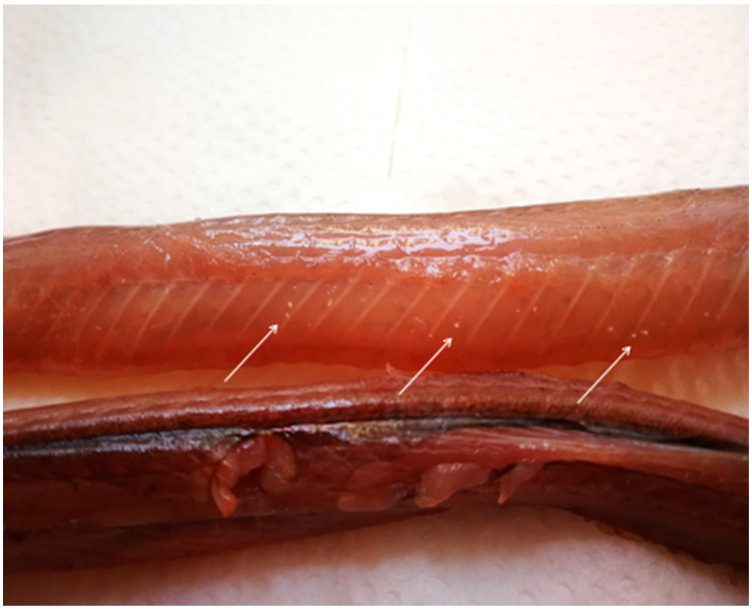
Trematodes mesocercariae in grass snake muscles (by E. Bilska-Zając).

**Figure 6:**
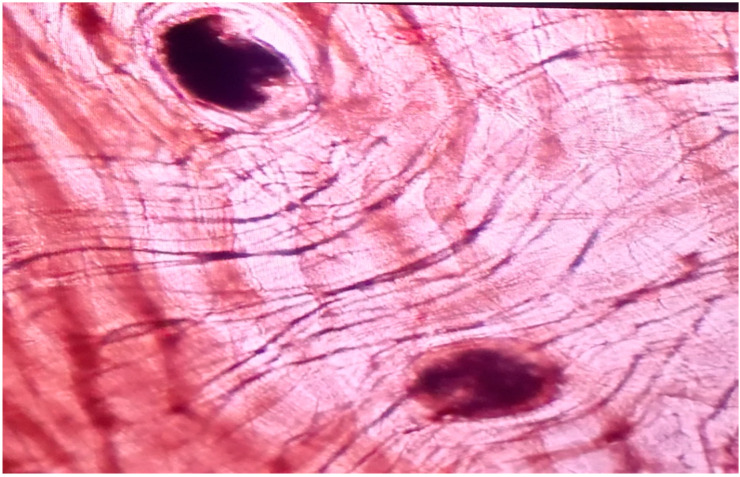
Trematode mesocercariae in snake muscle tissue visualized by trichinoscope (magnification 40×) (by E. Bilska-Zając).

The samples used in this study consisted of grass snakes road-killed along a 15 km-long asphalt forest road in Gostynińsko-Włocławski Landscape Park, central Poland (Figure 4), collected in October 2018 and in October 2019. A total of 51 snakes, including both recently-killed animals (a few hours) and dried carcasses (several hours), were collected and identified as European grass snakes based on morphological characteristics [6]. The snakes were photographed, measured, weighed and then eviscerated. The subcutaneous tissue was carefully screened for the presence of parasites (Figure 5), and the gut, intestine and muscles were checked under a stereomicroscope (Figure 3 and Figure 6). Afterward, individual muscle samples weighing 10-30 g, depending on snake size, were cut into slices about 1 cm in diameter and specifically investigated for *A. alata* mesocercariae by the AMT method [22]. The samples previously analyzed by AMT were also tested by the magnetic stirrer method (MSM) routinely used for *Trichinella* spp. detection (Commission Regulation (EC) 2020/1478) [41]. All isolated parasites (nematodes, trematodes and tapeworms) were counted, transferred to Eppendorf tubes and stored in 96% ethanol prior to DNA extraction and molecular identification [22,42,43,44]. The remaining helminths that were not used for DNA isolation were collected in 96% alcohol and placed in conditions of −18 degrees Celsius.

### 4.2. Statistical Analysis

A statistical analysis was performed including the calculation of the median and prevalence. These values were calculated in a Microsoft Office Excel spreadsheet (Table 1). The distribution of quantitative variables was tested by the Shapiro–Wilk test (using Statistica 9.1 Stat Soft) and the normality hypothesis of the data was rejected.

### 4.3. Species Identification by PCR Assay

One to five specimens, depending on parasite burden, were tested in PCRs. The DNA extraction was carried out from a single parasite using a DNA IQ System (Promega, Madison, WI, USA) according to the manufacturer’s protocol. In particular, to test morphology-based species identification, partial sequences of three genes were used: the mitochondrial cytochrome c oxidase subunit 1 (COI) gene and the nuclear genes for 18S ribosomal rRNA (18S) and 28S ribosomal rRNA (28S) genes. Trematodes were identified by amplification of the 18S gene (232 bp) [22] and nematods by the COI gene (207 bp) [43]. Additionally, *A. alata* was identified by the amplification of other COI fragments (450 bp) [22]. DNA form tapeworms was used to amplify the 28S gene [44] using LSU-5 and 1500R primers (~1400 bp).

Amplification products were obtained according to assumptions, and next subjected to standard Sanger sequencing; reverse and forward sequences were analyzed in the Geneious R7 program. Consensus sequences were compared with GenBank data by the BLAST [45] nucleotide algorithm to confirm species identification. 

## 5. Conclusions

This study on grass snakes of Gostynińsko-Włocławski Park shows that they are an excellent vector of *A. alata*, being an important source of infection for other susceptible animals living in the same area. As paratenic hosts with a parasite prevalence of more than 60%, grass snakes can play an important role in maintaining the life cycle of this trematode, particularly when they became prey to *A. alata*’s final hosts, such as foxes, who contribute to spreading this parasite over larger areas. The fact that infected grass snakes can be eaten by wild boars, causing an increase in the prevalence of *A. alata* in the wild boar population, is of particular interest. From the epidemiological point of view, since wild boar meat is intended for human consumption, the high prevalence of *A. alata* in grass snakes could have an indirect, but remarkable, effect on increasing the risk of human alariosis.

The occurrence of the other trematodes, nematodes and cestodes present in grass snakes indicates the role of this free-living animal as a huge reservoir for all these parasites. This should be borne in mind, especially when zoonotic parasites are discovered, posing a risk to human health.

## Figures and Tables

**Table 1:** Helminths prevalence in grass snake specimens collected in Gostynińsko-Włocławski Landscape Park, including mixed invasions.

Class of Helminth	Species of Helminth	No. of Infected Snakes [Prevalence (%)]	[Median; Intensity * (Range)]
**Trematodes**	*A. alata*	25/51 [49]	[65; 13–319]
	*A. alata + C. spathula*	2/51 [3.9]	[46; 42–50]
	*A. alata + C. spathula + S. falconis*	1/51 [1.9]	[125; 4–125]
	*A. alata + S. falconis*	2/51 [3.9]	[218; 70–366]
	*C. spathula*	5/51 [9.8]	[162; 5–320]
	*C. spathula + N. attenuatum*	1/51 [1.9]	[10; 1–10]
	*C. spathula + S. falconis*	7/51 [13.7]	[34; 3–335]
	*S. falconis*	2/5 [13.9]	[9.5; 4–15]
	*N. attenuatum*	1/51 [1.9]	[7.5; 1]
	Unidentified	4/51 [7.8]	[7.5; 1]
**Nematodes**	*C. vulpis*	4/51 [7.8]	[3.5; 1–17]
**Cestodes**	*Ophiotaenia*	1/51 [1.9]	[1; 1]

* Intensity- number of larvae found in grass snakes.

## References

[1] Sulgostowska T. (1971). Some parasites of grass snake *Natrix natrix* (L.) from Warszawa environment. State Sci. Publ..

[2] Borkovcova M., Kopřiva J. (2005). Parasitic helminths of reptiles (*Reptilia*) in south Moravia (Czech Republic). Parasitol. Res..

[3] Isaac L.A., Gregory P.T. (2004). Thermoregulatory behaviour of gravid and non-gravid female grass snakes (*Natrix natrix*) in a thermally limiting high-latitude environment. J. Zool..

[4] Mihalca A.D., Miclaus V., Lefkaditis M. (2010). Pulmonary Lesions caused by the Nematode *Rhabdias fuscovenosa* in a Grass Snake, *Natrix natrix*. J. Wildl. Dis..

[5] Petrova I.V., Chizhikova N.A., Pavlov A.V. (2010). Microclimatic Conditions of Environment in Termobiology of *Natrix natrix*. Uchenye Zap. Kazan. Univ.-Seriya EStestvennye Nauk..

[6] Herczek A., Gorczyca J. (2004). Płazy i gady Polski. Wydaw. Kubajak.

[7] Juszczyk W. (1987). Płazy i gady krajowe. Cz. 3. Gady-Reptilia. Wars. State Sci. Publ..

[8] Erdoğan D., Tosunoğlu M. (2017). Plasma Biochemical parameters of *Natrix natrix* (Linnaeus, 1758; Squamata: Natricidae) population in Çanakkale. Russ. J. Herpetol..

[9] Brown P.R. (1991). Ecology and Vagility of the Grass Snake, *Natrix natrix* Helvetica Lacepede. Ph.D. Thesis.

[10] Filippi E., Luiselli L. (2002). Negative effect of the wild boar (Sus scrofa) on the populations of snakes at a protected mountains forest in central Italy. Ecol. Mediterr..

[11] Möhl K., Grosse K., Hamedy A., Wüste T., Kabelitz P., Lücker E. (2009). Biology of *Alaria* spp. and human exposition risk to Alaria mesocercariae—A review. Parasit. Res..

[12] Beaver P.C., Little M.D., Tucker C.F., Reed R.J. (1977). Mesocercaria in the Skin of Man in Louisiana. Am. J. Trop. Med. Hyg..

[13] Zając M., Wasyl D., Różycki M., Bilska-Zając E., Fafiński Z., Iwaniak W., Krajewska M., Hoszowski A., Konieczna O., Fafińska P. (2016). Free-Living snakes as a source and possible vector of *Salmonella* spp. and parasites. Eur. J. Wildl..

[14] Gradba-Kazubska B. (1961). Parasites of the grass snake *Natrix natrix* (L.) in Poland. Wiad. Parazytol..

[15] Lewin J. (1992). Parasites of the water snake, *Natrix natrix* L., in Poland. Act. Parasitol..

[16] Mihalca A.D., Gherman C., Ghira I., Cozma V. (2007). Severe granulomatous lesions in several organs from *Eustrongylides* larvae in a free-ranging dice snake, *Natrix Tessellata*. Vet. Pathol..

[17] Wójcik A.R., Grygon-Franckiewicz B., Zbikowska E. (2002). Current data of *Alaria alata* (Goeze, 1782) according to own studies. Vet. Med.-Sci. Pract..

[18] Takeuchi-Storm N. (2015). *Alaria alata* Mesocercariae among feral cats and badgers, Denmark. Emerg. Infect. Dis..

[19] Korpysa-Dzirba W., Różycki M., Bilska-Zając E., Karamon J., Sroka J., Bełcik A., Wasiak M., Cencek T. (2021). *Alaria alata* in Terms of Risks to Consumers’ Health. Foods.

[20] Chmurzyńska E., Różycki M., Bilska-Zając E., Karamon J., Cencek T. (2013). *Alaria alata*-Potential threat for humans, prevalence and diagnostic measures. Vet. Life.

[21] Karamon J., Sroka J., Dąbrowska J., Bilska-Zając E., Skrzypek K., Różycki M., Zdybel J., Cencek T. (2020). Distribution of Parasitic Helminths in the Small Intestine of the Red Fox (*Vulpes vulpes*). Pathogens.

[22] Bilska-Zajac E., Marucci G., Piróg-Komorowska A., Cichocka M., Różycki M., Karamon J., Sroka J., Bełcik A., Mizak I., Cencek T. (2021). Occurrence of *Alaria alata* in wild boars (*Sus scrofa*) in Poland and detection of genetic variability between isolates. Parasitol. Res..

[23] Strokowska N., Klich D., Bełkot Z., Wiśniewski J., Didkowska A., Chyla P., Anusz K. (2020). The occurrence of *Alaria alata* mesocercariae in wild boars (*Sus scrofa*) in north-eastern Poland. Int. J. Parasitol.-Parasites Wildl..

[24] Riehn K., Hamedy A., Grosse K., Zeitler L., Lücker E. (2010). A novel detection method for *Alaria alata* mesocercariae in meat. Parasit. Res..

[25] Fernandes B.J., Cooper J.D., Cullen J.B., Freeman R.S., Ritchie A.C., Scott A.A., Stuart P.F. (1976). Systemic infection with *Alaria Am.* (*Trematoda*). Can. Med. Assoc. J..

[26] McDonald H.R., Kazacos K.R., Schatz H., Johnson R.N. (1994). Two cases of intraocular infection with *Alaria* mesocercaria (Trematoda). Am. J. Ophthalmol..

[27] Lewin J., Grabda-Kazubska B. (1997). Parasites of *Vipera berus* L. in Poland. Act. Parasitol..

[28] Shimalov V., Shimalov V. (2000). *Helminth fauna of snakes* (*Reptilia*, *Serpentes*) in Belorussian Polesye. Parasitol. Res..

[29] Kirillov A., Kirillova N.Y. (2021). Helminth fauna of reptiles in the National Park «Smolny», Russia. Nat. Conserv. Res..

[30] Komorova P., Sitko J., Špakulová M., Hurníková Z. (2016). Intestinal and liver flukes of birds of prey (*Accipitriformes, Falconiformes, Strigiformes*) from Slovakia: Uniform or diverse compound?. Parasitol. Res..

[31] Sitko J.Z. (1998). Trematodes of birds of prey (*Falconiformes*) in Czech Republic. Helminthology.

[32] Taft S.J., Suchow K., Van Horn M. (1993). Helminths from some Minnesota and Wisconsin raptors. Proc. Helminthol. Soc. Wash.

[33] Borgsteede F.H.M., Okulewicz A., Zoun P.E.F., Okulewicz J. (2003). The helminth fauna of birds of prey (*Accipitriformes, Falconiformes and Strigiformes*) in the Netherlands. ACTA Parasitol..

[34] Sanmartin M.L., Alvarez F., Barreiro G., Leiro J. (2004). Helminth fauna of Falconiform and Strigiform birds of prey in Galicia, Northwest Spain. Parasitol. Res..

[35] Mortier J.R., Fina C.J., Edery E., White C.L., Dhumeaux M.P. (2018). Computed tomographic findings in three dogs naturally infected with *Crenosoma vulpis*. Vet. Radiol. Ultrasound.

[36] Tolnai Z., Széll Z., Sréter T. (2015). Environmental determinants of the spatial distribution of *Angiostrongylus vasorum*, *Crenosoma vulpis* and *Eucoleus aerophilus* in Hungary. Vet. Parasitol..

[37] Morandi B., Bertaso S., Conboy G., Gustinelli A., Galuppi R., Tosi G., Poglayen G. (2019). *Crenosoma vulpis* in red foxes (*Vulpes vulpes*) in Northern Italy. Parasitol. Res..

[38] Bihr T.P. (1998). Crenosoma vulpis and the Domestic Dog: A Study of Prevalence on Prince Edward Island and of New Diagnostic Approaches.

[39] Santoro M., Tkach V.V., Mattiucci S., Kinsella J.M., Nascetti G. (2011). *Renifer aniarum* (*Digenea: Reniferidae*), an introduced North American parasite in grass snakes *Natrix natrix* in Calabria, southern Italy. Dis. Aquat. Org..

[40] Ammann M., Chambrier A. (2008). *Ophiotaenia gilberti* sp. n. (*Eucestoda: Proteocephalidea*), a parasite of *Thamnodynastes pallidus* (*Serpentes: Colubridae*) from Paraguay. Rev. Suisse Zool. Ann. Soc. Zool. Suisse Mus. D’histoire Nat. Genève.

[41] Commission Implementing Regulation (EU) 2020/1478 of 14 October 2020 Amending Implementing Regulation (EU) 2015/1375 as Regards Sampling, the Reference Method for Detection and Import Conditions Related to *Trichinella* Control (Text with EEA Relevance) C/2020/6922. https://eur-lex.europa.eu/legal-content/EN/TXT/?uri=uriserv%3AOJ.L_.2020.338.01.0007.01.ENG&toc=OJ%3AL%3A2020%3A338%3ATOC.

[42] Blaxter M.L., De Ley P., Garey J.R., Liu L.X., Scheldeman P., Vierstraete A., Vanfleteren J.R., Mackey L.Y., Dorris M., Frisse L.M. (1998). A molecular evolutionary framework for the phylum Nematoda. Nature.

[43] Allen S., Greig C., Rowson B., Gasser R.B., Jabbar A., Morelli S., Morgan E.R., Wood M., Forman D. (2020). DNA Footprints: Using Parasites to Detect Elusive Animals, Proof of Principle in Hedgehogs. Animals.

[44] Olson P., Cribb T.H., Tkach V.V., Bray R.A., Littlewood D.T.J. (2003). Phylogeny and classification of the *Digenea* (*Platyhelminthes: Trematoda*). Int. J. Parasitol..

[45] https://blast.ncbi.nlm.nih.gov/Blast.cgi?PROGRAM=blastn&PAGE_TYPE=BlastSearch&LINK_LOC=blasthome.

